# Plant Responses to Biotic and Abiotic Stresses: Crosstalk between Biochemistry and Ecophysiology

**DOI:** 10.3390/plants11233294

**Published:** 2022-11-29

**Authors:** Muhammad Iftikhar Hussain, Adele Muscolo, Mukhtar Ahmed

**Affiliations:** 1 Department of Plant Biology & Soil Science, Universidad de Vigo, Campus Lagoas Marcosende, 36310 Vigo, Spain; 2 Department of Agriculture, Mediterranea University, Feo di Vito, 89122 Reggio Calabria, Italy; 3 Department of Agricultural Research for Northern Sweden, Swedish University of Agricultural Sciences, 90183 Umeå, Sweden; 4 Department of Agronomy, Pir Mehr Ali Shah Arid Agriculture University, Murree Road, Rawalpindi 46300, Pakistan

Biotic and abiotic stresses, such as drought, salinity, extreme temperatures (cold and heat) and oxidative stress, are often interrelated; these conditions singularly or in combination induce cellular damage. For example, severe stresses during critical growth phases may directly result in mechanical damage and changes in the synthesis of macromolecules in cellular settings. In addition, these stresses often lead to oxidative damage and involve the formation of reactive oxygen species (ROS) in plant cells. Usually, plants have mechanisms to reduce their oxidative damage via the activation of antioxidant enzymes and the accumulation of compatible solutes that effectively scavenge ROS. However, if the production of activated oxygen exceeds the plant’s capacity to detoxify it, deleterious degenerative reactions do occur, the typical symptoms being loss of osmotic responsiveness, wilting and necrosis. Given that plants face stressful conditions, imposed by changing environmental conditions that affect their growth and development during their whole life cycle, plants have to be able to perceive, process and translate different stimuli into adaptive responses.

The current Special Issue in *Plants* aims to analyze, from a multi-perspective approach (ranging from gas exchange, metabolomics, proteomics, isotopic and genomics, etc.), drivers (e.g., trait selection, phenotypic plasticity) and specific strategies used by the plants at physiological and molecular levels for their better adaptations to stressful growth conditions.

In total, 20 manuscripts (research and review) are included in this Special Issue. Furthermore, this Special Issue presents research findings in various experimental models (crops, fruit trees, legumes and halophytes) and areas ranging from cellular to ecophysiological and biochemical aspects.

Abideen et al. [1] used *Phragmites karka* to investigate the potential effects of salinity (control, 100 and 300 mM NaCl in a nutritional solution) and drought (at 50 percent water-holding capacity) and studied the correlations between stress tolerance, photosynthetic processes, biomass and ethanol output. They further discuss that plants exhibit an efficient photosynthetic system to grow in salty and dry environments, making it a viable crop for biofuel production.

Badar et al. [2] studied how to reduce the dangers of pharmaceutical pollution in the environment and investigated the bioremediation capability of edible crops and their associated microbial communities to successfully remove these pollutants. They tested paracetamol, which is also known as acetaminophen, at three concentrations (50, 100 and 200 mg/L) in terms of absorption, transport, accumulation and degradation in various organs of spinach (*Spinacia oleracea*) under controlled laboratory settings. Growth and photosynthetic machinery of the plants was negatively impacted by rising paracetamol stress levels. LC-MS data showed the drug absorption and translocation from roots to aerial parts and drug breakdown after eight days. Several bacterial strains (Burkhulderia, Sphingomonas, Pseudomonas, Staphylococcus, Stenotrophomonas and Kocuria) were isolated from spinach shoots and roots.

Hussain et al. [3] studied the rehabilitation of salt-degraded marginal soils through the selection and assessment of crop cultivars that can withstand salt stress. They evaluated the effects of different salinity levels (0, 7 and 14 dS m^−1^) on six barley genotypes (113/1B, 59/3A, N1-10, N1-29, Barjouj and Alanda01) and evaluated different physiological, biochemical and stable isotopic responses. All measured plant traits responded to salt in a genotype-specific way. They showed that the genotypes (Barjouj and Alanda01) proved their suitability under the sandy desert soils of Dubai, UAE, as they exhibited higher grain yield, while 113/1B and Barjouj have greater grain protein content. The present research demonstrated that saline marginal sandy desert soils could support the cultivation of salt-tolerant barley genotypes for food and nutrition security as well as for the rehabilitation of marginal lands.

Riaz et al. [4] assessed the phytochemical potential of *Ziziphus* species, i.e., *Z. jujuba*, *Z. mauritiana*, *Z. spina-christi* and *Z. nummularia*, from desert environments. Leaf length, leaf width, leaf area and leaf petiole length were higher in *Z. jujube*, while *Z. mauritiana* exhibited higher dry biomass. *Z. jujube* had the largest fruit length, fruit stalk length, fruit diameter, fruit width, fruit area, seed length and seed diameter, while *Z. nummularia* had the highest fruit dry weight and widest seeds. Secondary metabolites were found in the fruits and leaves of *Ziziphus* species, including phenol, flavonoids and antioxidant activity. The highest levels of fruit phenols (304.4 mg GAE/100 g), leaf phenols (314.2 mg GAE/100 g), fruit flavonoids (123.7 mg QE/100 g) and leaf flavonoids (113.4 mg QE/100 g) were all accumulated by *Z. nummularia*. Moreover, irrigated and drought plantations led to a significant variation in morphological, fruit characteristics and phytochemical constituents that might be useful for future production technologies for this medicinal plant.

Hussain et al. [5] evaluated lowland rice genotypes under well-watered (WW) and terminal water stress (TWS) for improving drought stress and yield stability. Genotypes Look Pla and Lep Nok were found to be stress tolerant, whilst genotypes Chor Lung, Hom Nang Kaew and Hom Chan were found to be moderately tolerant genotypes. Genotypes Hom Pathum, Sang Yod, Dum Ja and Pathum Thani-1 were found to be extremely stress tolerant and relatively high yielding. Different stress-tolerance metrics, such as the stress-tolerance index (STI), the geometric mean productivity (GMP), the mean productivity index (MPRO) and the harmonic mean index (MHAR), showed significant and favorable correlations with GY during WW.

Ndiate et al. [6] demonstrated, in a greenhouse study, the impact of biochar (5%), arbuscular mycorrhizal fungus (20 g/pot, AMF) and biochar + AMF on maize (*Zea mays* L.) plants grown under salt stress (0, 50, 100, and 150 mM NaCl), to describe the mitigating technique against salinity. Plant height and fresh weight were decreased by 17.84% and 39.28%, respectively, compared to the control after 90 days of treatment with 100 mM NaCl. The growth parameters rose by 22.04%, 26.97%, 30.92% (height) and 24.79%, 62.36% and 107.7% (fresh weight) when the saline-treated soil (100 mM NaCl) was supplemented with AMF, biochar and biochar + AMF, as compared to control. The biochar + AMF enhanced plant nutrient uptake, (ii) improving soil nutrient content, (iii) increasing antioxidant enzyme activity and (iv) improving the contents of palmitoleic acid (C16:1), oleic acid (C18:1), linoleic acid (C18:2) and linolenic acid (C18:3). They concluded that biochar and AMF addition to saline and alkaline soils can successfully reduce abiotic stress and enhance plant development.

Elkelish et al. [7] investigated cysteine (Cys) (25 and 50 ppm as a foliar application) and lipoic acid (ALA) (0.02 mM, grain dipping pre-cultivation treatment) under water deficit and well-watered irrigation (100% and 70% of the required dose). The deficit irrigation increased cellular oxidative damage via increased malondialdehyde (MDA) level and hydrogen peroxide (H_2_O_2_). Superoxide dismutase (SOD), ascorbate peroxidase (APX), catalase (CAT) and peroxidase (POX), osmolytes and chlorophyll (Chl) were among the enzymatic antioxidants that benefited from Cys administration. The ability of the plant to scavenge reactive oxygen species (ROS), leaf relative water content (RWC), grain number, total grain yield, weight of 1000 kernels and gluten index was improved. Additionally, heatmap plot analysis uncovered numerous significant connections between the various characteristics that were examined, which may be explored.

El-Serafy et al. [8] showed that hydro-priming and halo-priming with silicon (Si) and silicon nanoparticles (SiNPs) can improve salinity tolerance of the ornamental plant *Lathyrus odoratus*. They highlighted that halo-priming with Si or SiNPs increased Lathyrus seedling salt-stress tolerance. This effect was confirmed using seawater treatments, which improved the germination percentage, seedling growth and activation of the antioxidant machinery, which detoxifies reactive oxygen species (ROS).

Alnusairi et al. [9] investigated the effect of exogenously applied nitric oxide (NO) (50 μM and 100 μM) in protecting wheat plants from NaCl-induced oxidative damage by modulating protective mechanisms. They showed that the exogenous-sourced NO at both concentrations up-regulated the antioxidant system for averting the NaCl-mediated oxidative damage on membranes. Enhancing salt tolerance by NO was concomitant with an obvious down-regulation in the relative expression of SOS1, NHX1, AQP and OSM-34, while D2 protein was up-regulated.

Farooq et al. [10] evaluated total carbon (TC), total nitrogen (TN) and isotopic natural abundance of C (δ^13^C) and N (δ^15^N) in soil and foliage of coniferous plantation (CPF), natural broadleaved forest (NBF) and mixed-forest stands at three different soil depths (i.e., 0–10, 10–20 and 20–40 cm) and how soil-available nutrients are affected by different forest types. Results showed that soil nutrient availability was higher in mixed forests. The findings provided evidence that forest type and soil depth alter TC, TN and soil δ^15^N, whereas δ^13^C was only driven by soil depth. Moreover, plantations led to a decline in soil-available nutrient content compared with NBF and mixed-forest stands.

Farooq et al. [11] evaluated the effects of intercropping of peanut (*Arachis hypogaea* L.) with tea plants (*Camellia oleifera*), in comparison with the mono-cropping of tea and peanut. Soil health and fertility were examined. The results showed that the intercropping enhanced soil nutrient status and positively impacted soil conservation, increasing soil organic carbon (SOC), soil nutrient availability and soil enzymatic responses at different soil depths.

Rehman et al. [12] evaluated the morpho-physiological traits of two spring wheat cultivars (Millet-11, Punjab-11) and two advanced lines (V-07096, V-10110) exposed to terminal heat stress under late sowing. Results showed that improved grain yield was associated with the highest chlorophyll contents, showing stay green characteristics with maintenance of high photosynthetic rates and cooler canopies under late sowing and revealed that advanced lines and Punjab-11 with heat-adaptive traits could be promising sources for further use in the selection of heat-tolerant wheat genotypes.

Umnajkitikorn et al. [13] evaluated the potential of using elevated nitrogen priming prior to water shortage to mitigate plant stress through nitric oxide accumulation. Results indicated that plants primed with nitrogen possessed a higher photosynthetic rate, relative water content, electrolyte leakage and lipid peroxidation under water-deficit conditions, compared to control plants. The induction of water-deficit tolerance was supported by the activation of the antioxidant-defense system, induced by the accumulation of nitric oxide in leaves and roots of rice plants.

Hussain et al. [14] investigated the salinity-tolerance mechanisms of six contrasting quinoa cultivars belonging to the coastal region of Chile using agro-physiological parameters. Results suggested that all measured plant traits, except for C:N ratio, responded to salt in a genotype-specific way. Results indicated that the genotypes (Q21 and AMES13761) proved their suitability under sandy desert soils of Dubai, UAE, as they exhibited higher seed yield, while NSL106398 showed a higher seed protein content. The present research highlights the need to preserve quinoa biodiversity for a better seedling establishment, survival and stable yield in the sandy desert UAE environment.

Hassan et al. [15] evaluated the impact of various Cd concentrations (0, 5, 25, 50 and 100 M) on physiological and biochemical parameters in two sorghum (*Sorghum bicolor* L.) cultivars—JS-2002 and Chakwal Sorghum. The Cd absorption was increased in both cultivars while Cd uptake in JS-2002’s leaf, stem and root was greater than that of Chakwal Sorghum. The superoxide dismutase (SOD), peroxidase (POD) and catalase activities were also lowered by Cd stress at higher levels (50 and 100 M). Results showed that JS-2002 had a higher Cd tolerance.

Hussain et al. [16] investigated the phenolic compound and flavonoid composition and allelopathic effects of an aqueous extract of aerial parts from *Acacia melanoxylon* R. Br. on seedling growth and plant biomass of the general biotest species, lettuce (*Lactuca sativa*). The acacia flower aqueous extract (AFE) and phyllodes aqueous extract (APE) reduced the leaf fresh weight, leaf dry weight, root fresh weight and root dry weight in lettuce. The mean root length decreased by 37.7% and 29.20%, following treatment with Acacia flower extract (AFE) at a concentrations of 75% and 100%, respectively. In total, 13 compounds of gallic acid, protocatechuic acid, p-hydroxybenzoic acid, p-hydroxybenzaldehyde, vanillic acid, syringic acid, p-coumaric acid and ferulic acid were among the phytochemical substances found from both parts. Rutin, luteolin, apigenin and catechin are some of the principal flavonoid chemicals found and are potential causes of the allelopathic effects of floral and phyllodes extracts from *A. melanoxylon*.

Hussain et al. [17] studied allelopathy, which is an ecological phenomenon that occurs when biomolecules from various crops, cultivated plants and bacteria or fungi are produced and released into the soil rhizosphere and have an impact on nearby species. Sorghum allelopathy has been utilized in relation to green manure, crop rotations, cover crops, intercropping and mulching, plant aqueous extracts or powder. From various plant tissues of sorghum and root exudates, a variety of allelochemicals, including benzoic acid, p-hydroxybenzoic acid, vanillic acid, ferulic acid, chlorogenic acid, m-coumaric acid, p-coumaric acid, gallic acid, caffeic acid, p-hydroxibenzaldehyde, dhurrin and sorgoleone, was identified. Among them, sorgoleone has been studied for its mode(s) of action, specific activity, selectivity, release in the rhizosphere, uptake in vulnerable species and translocation. The importance of sorghum allelopathy as an ecological tool for managing weeds is discussed in this review, which also highlights the most recent discoveries regarding the allelochemicals found in sorghum, their mechanisms of action and their place in the environment.

Khan et al. [18] reported that the global temperature has been steadily rising at a pace of 0.15–0.17 °C every decade. Therefore, measures for thermotolerance are required to maintain crop output under increased temperatures. This review was carried out with the goal of providing information on the wheat reaction in three research fields, including physiology, breeding and genetic advancements. At the heading, anthesis and grain-filling stages of wheat growth, the ideal temperatures are 16 ± 2.3 °C, 23 ± 1.75 °C and 26 ± 1.53 °C, respectively. The high temperature has a negative impact on the phenology, growth and development of the crop. The pollen viability, seed germination and embryo development are all slowed down by the high temperature before anthesis. The accumulation of starch granules, stem-reserve carbohydrates and photosynthate translocation into grains is reduced by the high post-anthesis temperature. The reviewed work showed that the genotypes with higher levels of proline, glycine betaine, heat-shock protein expression, stay green and antioxidant enzyme activity, specifically catalase, peroxidase, super oxide dismutase and glutathione reductase, can tolerate high temperature effectively by supporting cellular physiology.

Raza et al. [19] demonstrated that the maize–soybean intercropping system has many persistent constraints, including lodging, which pose severe limitations to the development and sustainability of this cropping system. The lodging phenomenon is influenced by a variety of morphological and anatomical traits. Because of the shading from maize, soybean stems develop a shade-avoidance response, which causes stem elongation and significant lodging. The primary agro-techniques needed to investigate lodging in the maize–soybean intercropping system for sustainable agriculture, however, have not yet been clearly defined. The present review suggests that controlling lodging requires a variety of strategies, including agronomic, chemical and genetic ones, that could be useful in lowering lodging hazards in the maize–soybean intercropping system. Therefore, further research is needed from agronomists, physiologists, molecular biologists and breeders to address this difficult issue.

Finally, we encourage readers to view the articles published in this Special Issue of “Plant Responses to Biotic and Abiotic Stresses: Crosstalk between Biochemistry and Ecophysiology”.
